# Senses and meanings attributed to Hope by children with orofacial clefts and their family caregivers*

**DOI:** 10.1590/1980-220X-REEUSP-2025-0561en

**Published:** 2026-08-03

**Authors:** Mariana Martire Mori, Camila Moraes Garollo Piran, Alana Vitória Escritori Cargnin, Armandos dos Santos Trettene, Bianca Machado Cruz Shibukawa, Zaida Borges Charepe, Marcela Demitto Furtado

**Affiliations:** 1 Universidade Estadual de Maringá, Programa de Pós-Graduação em Enfermagem, Maringá, PR, Brazil.; 2 Faculdade de Medicina de Bauru, Hospital de Reabilitação de Anomalias Craniofaciais, Bauru, SP, Brazil.; 3 Universidade Federal do Mato Grosso do Sul, Departamento de Enfermagem, Três Lagoas, MS, Brazil.; 4 Universidade Católica Portuguesa, Centro de Investigação Interdisciplinar em Saúde, Lisboa, Portugal.

**Keywords:** Child, Caregivers, Hope, Cleft Lip, Cleft Palate

## Abstract

**Objective:**

To understand the senses and meanings attributed to Hope by children with orofacial clefts and their family caregivers.

**Method:**

This is a qualitative, descriptive, and exploratory study, based on the Hope-Promoting Mutual Help Intervention Model and Symbolic Interactionism. It was conducted at an association in Paraná, with children aged five to ten years, with orofacial clefts, and with family caregivers of children under ten years old with the same condition. Data were collected through Dramatic Therapeutic Play and semi-structured interviews. Thematic analysis was of the deductive type, with the support of *Atlas.ti* ^®^.

**Results:**

Sixteen children with orofacial clefts and 26 family caregivers participated. For children, Hope was associated with the expectation of good things, including professional aspirations and a transformation of self-image. For caregivers, Hope was associated with inner strength and spirituality, essential for giving meaning to life.

**Conclusion:**

Hope is an essential dimension for children with orofacial clefts and their family caregivers in coping with rehabilitation, and is fundamental to the care process.

## INTRODUCTION

Hope is an essential factor in promoting mental health, capable of protecting individuals from psychological suffering, promoting comfort, and improving quality of life^(1)^. This is an important dimension in vulnerable contexts, especially those related to health problems^(2)^. Likewise, it is an important dynamic force in promoting, maintaining, and sustaining life, capable of protecting people from anxiety and suffering^(3)^.

Hope refers to a dynamic resource built through social relationships and bonds between family members, and, in the context of chronic pediatric diseases, Hope can be influenced by social support, access to information, and the evolution of the health status^(4)^. Thus, in contexts of chronic conditions marked by uncertainty and prolonged treatment periods, Hope can be strengthened or weakened through the experiences of children and their caregivers^(3)^. In children’s health, Hope is a resource that positively influences coping with adversity, manifested through the establishment of goals that children strive to achieve, positive behaviors that contribute to well-being and social interactions, or individual beliefs^(5)^.

Orofacial cleft is one of the most frequent congenital malformations, affecting approximately one in every 650 live births^(6)^. This is a condition resulting from alterations in facial development, the severity of which varies and can compromise essential functions such as feeding, speech, and the child’s social development^(7)^. The psychological challenges faced by children with this malformation are numerous, mainly related to appearance and self-esteem, and are associated with the social and family environment in which they are embedded^(8)^. Considering the symbolic value of appearance in social interactions, stigmas and insecurities regarding children’s appearance can interfere with the development of positive expectations for the future^(9)^.

In addition to compromising children’s health, orofacial clefts have the potential to alter the emotional well-being of their family caregivers, as they share the children’s experiences and offer support and assistance throughout the rehabilitation process, which is marked by surgical procedures, speech rehabilitation, psychological and oral health care^(10)^.

Women responsible for children with cleft palate often experience intense feelings of sadness^(7)^. This emotional experience can be explained by several factors, including clinical interventions, the constant demands of caring for a child with specific needs, social judgment related to the child’s appearance, the limited information available to the general public about the condition, as well as the limited availability of support networks and social assistance^(10)^.

Thus, according to the Hope-Promoting Mutual Help Intervention Model (*MIAMPE*), support and mutual help are factors that promote mental health and Hope in people experiencing a chronic condition^(3)^. In addition, Symbolic Interactionism (SI) reveals that social interactions are relevant to the shared construction of meaning among individuals^(11)^. From this perspective, it becomes essential to consider the child’s ability to construct meaning about Hope and orofacial clefts. Therefore, addressing Hope for children with orofacial clefts and their family caregivers is a way to understand the complexity of the senses and meanings of Hope, providing greater reliability in the findings.

In this regard, considering that orofacial cleft is a chronic condition with an arduous rehabilitation process, it is necessary to understand the meaning and role of Hope for children with this malformation and their family caregivers, to include, in addition to clinical conditions, emotional and social factors in the treatment plan^(12)^. By integrating Hope into therapeutic planning, care is broadened beyond biomedical interventions, being recognized as a supportive resource in the rehabilitation process and a strong indicator of emotional well-being. Furthermore, the lack of studies addressing Hope in children with orofacial clefts and their caregivers should be highlighted, suggesting a knowledge gap. Therefore, the following question arises: What are the senses and meanings attributed to Hope by children with orofacial clefts and their family caregivers? As a consequence, this study aims at understanding the senses and meanings attributed to Hope by children with orofacial clefts and their family caregivers.

## METHOD

### Design of Study

This qualitative, descriptive, and exploratory study is anchored in *MIAMPE*, which considers Hope in all its breadth as essential for the promotion, maintenance, and sustenance of life, and protection against suffering^(3)^. It was also based on SI, a theory that considers symbols, meanings, and social interactions to be important for the construction of social reality, which can be shaped according to experience^(11)^.

The selection of *MIAMPE* is warranted as it considers Hope in its full dimension, as an essential force in confronting suffering, especially in contexts of vulnerability^(3)^. SI allows us to understand how the meanings of Hope are constructed in social interactions and mediated by symbols^(11)^, fundamental for subjective investigations.

The recommendations in the *Consolidated criteria for reporting qualitative research (COREQ)* guide were followed to ensure greater transparency in the study^(13)^.

### Local

The study was conducted at a support association that provides care to children and adolescents with orofacial clefts and their families, located in the northwest of the state of Paraná, Brazil, which aims to rehabilitate this population. The care provided at the institution is free, linked to the Brazilian Public Health System (*SUS*), and directed to 80 municipalities, serving approximately 500 people and their families.

### Participants and Selection Criteria

The sample size was determined by convenience, inviting children with orofacial clefts, between five and ten incomplete years of age, and family caregivers of children under ten years of age with orofacial clefts, who attended appointments at the service between November 2024 and April 2025, to participate in the study. Among those invited to participate, there were no refusals. The age range for children was chosen because it coincides with the period in which children have the ability to represent the reality they experience, a process marked by symbolic play^(14)^. Conversely, family caregivers of children under ten years of age were considered because they have different experiences considering the child’s rehabilitation phase, which would allow for a more comprehensive understanding of Hope.

For children, the eligibility criteria adopted were: children with isolated orofacial cleft (not associated with other syndromes), within the established age range, and who regularly attend the service according to the schedules established by the team. Regarding family caregivers, the following criteria were considered: being the primary family caregiver and residing in the same household as the child, considering that daily interaction allows for monitoring of the rehabilitation routine and fosters a deeper understanding of the factors that influence the building of hope in this process; actively participating in the treatment; and being over 18 years of age. For both groups, those who presented with a cognitive deficit, attested by a physician, that influenced their understanding of the questions were excluded. It should be noted that family caregivers and children from various family units were addressed.

### Data Collection

The data were collected between November 2024 and April 2025 by the principal investigator, a nurse and master’s student, who has experience with the data collection technique. The researcher has been involved in a research project at the institution for five years and is familiar with the service’s routine; however, she does not provide direct assistance to the children and their families. The director of the institution was contacted to determine the best way to conduct the research, so as not to disrupt the routine of patient care. It is important to note that participants were selected based on scheduled appointments, respecting the established eligibility criteria.

Data collection took place at the association itself, in a private room and in a calm environment, without interruptions, in the interval between consultations, on an individual basis. The children were approached only once each, in a Dramatic Therapeutic Play (DTP) session guided by the question: “Let’s play pretend, like a child who has an orofacial cleft and is searching for Hope?” The DTP is a strategy that facilitates bonding between the child and the researcher, and facilitates the child’s verbal expression^(14)^.

**Figure 1:**
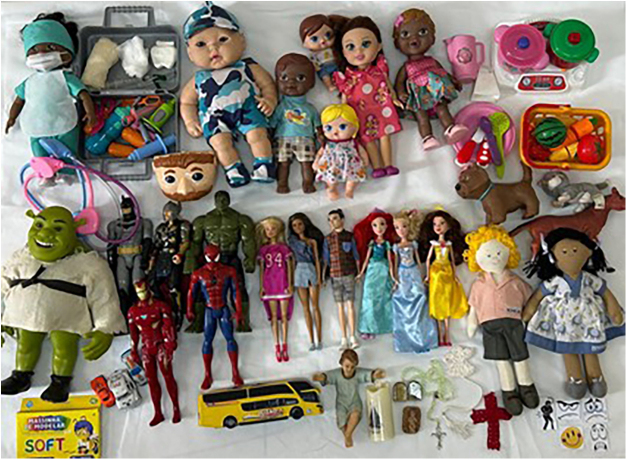
Toys used in the research.

Source: Authors, 2025.

Various materials were offered, including dolls to represent family, healthcare professionals, animals, toys for domestic and hospital use, superheroes and princesses, emotion stickers, dolls with cleft lip and palate, toy cars, buses, modeling clay, and symbolic objects related to spirituality (rosary, cross, angel, candle, Bible, and image of Baby Jesus), which were selected due to their sociocultural recognition as symbols frequently associated with expressions of faith. The children were allowed to choose those with whom they felt most represented (Figure 1). The toys were collected at the end of the session. Each session lasted 50 minutes and was recorded using audiovisual means (video and audio), with the permission of the guardians.

For family caregivers, a semi-structured interview based on *MIAMPE* was used, guided by the question “What is Hope for you?”, and other auxiliary questions. The interviews were conducted only once with each caregiver, were recorded, lasted approximately 20 minutes each and, at the end, the statements were validated through an oral summary presented to the participant, who was allowed to remove or add content.

The data collection instruments were validated, in a simplified manner, by three Nursing PhD judges who have expertise in the area of child, adolescent, and family health. A pilot test was conducted with four children and four caregivers, and, as no need to change the instruments was observed, these interviews were included in the final sample. Data collection was completed when the researcher observed that the data already answered the research objective, through the analysis and depth of the statements^(15)^.

### Data Analysis

The interviews were transcribed in full as they were conducted. The material was imported to the software *Atlas.ti* ^®^, a tool to assist in data organization, coding, and systematization. The analysis was conducted according to the assumptions of deductive thematic analysis, following these steps: I- The material was transcribed and read in depth; II- Codes were created systematically; III- Themes were searched using the identified codes; IV- The themes were reviewed and a thematic map was created in the software; V- The themes were named; VI- The speeches were analyzed again for checking correspondence to the research question^(16)^.

To broaden the understanding of Hope and validate the data, data triangulation was performed by collecting information from two groups: children with orofacial clefts and their family caregivers. This allowed for a comparison of findings and enriched the analysis^(17)^.

The process underwent validation by three PhDs in nursing, in which, after the initial coding performed by the principal investigator, the codes and themes, along with the participants’ statements, were shared to ensure that the result faithfully reflected their statements. The analysis was discussed in meetings, where convergences and divergences in the interpretation of the data were examined. In cases of discrepancy, the material was reviewed jointly until a consensus among the researchers was reached.

### Ethical Aspects

The study was conducted in accordance with the guidelines of Resolution No. 466/12 of the National Health Council/Ministry of Health, and was approved by the Human Research Ethics Committee of the university, under opinion No. 7.041.250/2024.

The research was explained to the participants using accessible language. The Free and Informed Consent Form was applied to the family caregivers. For the children, the Free and Informed Assent Form was used, which was adapted to age-appropriate language, including graphic elements to facilitate understanding.

To ensure the preservation of the participants’ identities, the children were named according to the initial “C” for child, followed by the order of the interviews and their age, while the family caregivers were identified according to their degree of kinship with the child, followed by the order of the interviews.

## RESULTS

In total, 16 children participated in the study. There was no predominance between the sexes, with eight girls and eight boys; the age ranged from five to nine years; and regarding the type of cleft, eight were pre-foraminal and eight transforaminal.

Concerning the caregivers, 26 participated, including 19 mothers, two fathers, two aunts, and three grandmothers. The age ranged from 20 to 75 years, with 92% (n = 24) being female and 65% (n = 17) married.

**Figure 2:**
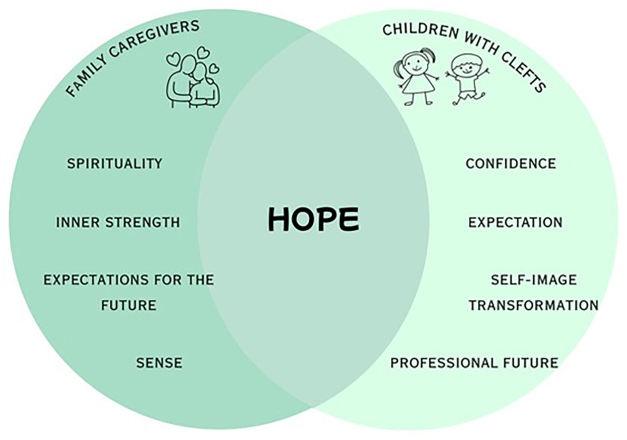
Meanings of Hope for children with orofacial clefts and their family caregivers.

Source: Research data, 2025.

It was observed that Hope has different meanings for children with orofacial clefts and their family caregivers, and these can be understood through Figure 2. In both groups, Hope was related to the future; however, for the children, the future was related to their professional future. In contrast, for caregivers, the future is seen as the fulfillment of dreams and a belief in the child’s potential. The transformation of self-image was reported as significant for the children. For family caregivers, Hope was more related to an inner strength based on faith and spirituality.

Two thematic categories emerged from data analysis: 1. Meanings attributed to Hope by children with orofacial clefts, and 2. Meanings of Hope for Family Caregivers of Children with Orofacial Clefts.

### 1. Meanings Attributed to Hope by Children with Orofacial Clefts

The meanings attributed to Hope by children with orofacial clefts were explored by mediation with toys. Hope was associated with the expectation of good things, overcoming sadness, and having confidence even in the face of adversity.

*[points to the bus, Hulk and Spider-Man] They’re toys, which is a good thing. Hope is when things get better.* (C7, 06 years)

*Hope is the last thing to die. You can’t lose hope while you still have your life. Almost everything gives me hope, well, almost everything gives me hope.* (C12, 09 years)

*[Playing dentist] May good things happen.* (C13, 08 years)

*It’s about finding something that’s lost, and also about feeling very sad and wanting to find something, wanting to do something.* (C16, 07 years)

The children attributed Hope to a desire for self-image transformation and the projection of future parents of children with clefts, reproducing the care they received. Furthermore, they attribute a negative meaning to adult life due to societal prejudice.

*When I get older I want the skin around my lips to come off, I don’t like it the way it is.* (C7, 06 years)

*Before going to work, I put on makeup to keep my face smooth and so this [scar] doesn’t show. What if the adults see and make fun of me? Because I’m not going to be a child anymore, so people won’t make fun of me. I imagine that in the future all my children will have mouths like that, I just want it to happen with a girl who will be my daughter because I’ll find it cute [picks up a doll to represent]. If I think she won’t like it, I’ll say that I had it too. My daughter, when I have her, what if she doesn’t like it? I’ll say, “It’s okay, you were born this way.” If she feels unwell, I’ll take her to the hospital and bring her here [to the association].* (C16, 07 years)

During playtime, the children with cleft lip and palate also expressed Hope related to their professional future, attributing meaning to the professionals responsible for their rehabilitation and wishing to perform the same role in the future. The senses emerged through toys when children selected dolls and medical instruments. These objects were used to represent everyday scenes experienced during rehabilitation.

*I’m playing doctor. My mom inspired me to be a doctor because she gave me a toy doctor set that had Barbie dolls, and I would do those Barbie things, and that’s how I got inspired to be a doctor.* (C1, 07 years)

*I want to be a teacher because I can help my students and I think it’s a good job. I wanted to be a doctor, but I can’t stand the sight of blood and injured people. I think it’s a good function because many people can be healed and won’t get sick again.* (C11, 08 years)

*I hope to at least become someone in life, because God willing, I’ll go to college. My mother tells me to start figuring out what I’m going to do. But everyone tells me to go to medical school, but I don’t really like it, I think.* (C12, 09 years)

Hope for children with cleft lip and palate has been attributed to the expectation of good things happening and support in overcoming sadness, permeated by a desire for transformation. Furthermore, it was also related to confidence in the future, connected to treatment and professional achievement, often inspired by the professionals who care for their own rehabilitation. Moreover, children want to replicate in the future the situations they experience during rehabilitation, such as support and care, which highlights the importance of good role models.

### 2. Meanings of Hope for Family Caregivers of Children with Orofacial Clefts

For family caregivers of children with cleft lip and palate, Hope evokes the meaning of inner strength, based on faith and spirituality. Furthermore, hope is related to things that people believe in, but don’t necessarily visualize.

*Hope, I think, is that if we trust, we can achieve things through our faith, not through what we see, but through what we believe.* (Mother 05)

*Hope is what makes us wake up every day, of having a better life, of being able to see our children grow up.* (Mother 06)

*It’s knowing that something will work out even when everything seems to be going wrong.* (Mother 16)

Caregivers associate hope with what gives meaning to the children’s lives and treatment, believing that they will have better days. Caregivers believe that Hope is essential to filling the inner void.

*We have to have hope in something in life, we have to have something to fight for. Having hope motivates me even more because believing makes me want to keep searching.* (Aunt 02)

*Hope is what drives us; if we have hope, we can have a better life and a better future. I believe that a person without hope is an empty person, because there is nothing to fill the void. Hope is a feeling, not something we can see or make concrete.* (Mother 05)

*I think this journey is very difficult, and if we don’t have hope, we won’t get anywhere.* (Mother 15)

Some family members attribute hope to the children’s future, recognizing their potential and providing encouragement. For them, Hope is to one day see the children rehabilitated and fulfilling their dreams.

*I say to him, ‘What do you want to be?’ We filled him with hope because he felt incapable, he would say, “I’m stupid.” I ask him, ‘What do you want to be?’, and he says he wants to be a police officer.* (Aunt 02)

*My hope for her future is to see my daughter fully rehabilitated, shouting and talking about this cleft lip and palate that is her story, showing everyone that a child with a cleft lip and palate can achieve anything they want, can be whoever they want to be.* (Mother 03)

*We think like this: I want you to be someone, whatever you want to be, you are capable of being, there are no limits, no boundaries, you have the ability and you can do it.* (Mother 05)

For family caregivers, Hope is necessary to face rehabilitation, supported by faith and spirituality. Additionally, it gives meaning to life and provides confidence in the complete rehabilitation of children and their dreams fulfilment.

## DISCUSSION

The sense and meaning attributed to Hope for children with cleft lip and palate and their family caregivers are dynamic, projected towards the future, related to social interactions, emotional bonds, care, spirituality, and the rehabilitation process. Hope takes on different roles for children and their family caregivers. The research results indicate that, although shared, Hope assumes distinct functions. For children, it was related to identity building, while for caregivers it is a resource for emotional support.

For children, hope translates into well-being, emotional support, and overcoming of sadness that may occur during the rehabilitation process. These results align with the assumptions of *MIAMPE*, which argues that Hope is a vital force that alleviates suffering and propels people in their pursuit of transformation^(3)^. Similarly, a study conducted with students in China showed that hope is related to good mental health, through the strengthening of psychological resilience in challenging contexts^(18)^.

Through play, the children represented their desire for a professional future and to perform important professions in their rehabilitation process, such as doctors, nurses, and dentists, giving new meaning to the care they receive from these professionals. The findings reinforce how meanings are socially constructed from lived experiences^(11)^.

However, contradicting the findings of this study, research using drawings, conducted with children from a Brazilian school, indicated that the profession of dental surgeon is viewed negatively by children, associated with expressions of fear and pain, and traits that suggest tension, anxiety, and negative emotions^(19)^. However, the experience with dental procedures differs among these children, since children with orofacial clefts view the profession as a possibility to improve their image and the alterations caused by the malformation.

The children also revealed problems with self-image and concern about prejudice in the future, in adulthood. Therefore, they consider Hope to be fundamental in facing the fear and insecurity experienced during the rehabilitation process. This role has also been demonstrated in other health contexts, such as in stroke patients, where Hope acts as a factor that positively influences resilience^(2)^.

For family caregivers of children with cleft lip and palate, Hope has emerged as an inner strength that gives meaning to life, sustained through spirituality. These perceptions confirm the assumptions of *MIAMPE*, in which spirituality is a pillar that sustains hope in the face of adversity^(3)^. These results are reinforced by a study conducted with caregivers of children with various chronic conditions, which identified that hope linked to spirituality is a motivational factor in their lives^(20)^.

This research highlighted the predominance of women, especially mothers, as the primary caregivers of children, emphasizing female centrality. Historically and culturally, caregiving is attributed as a female responsibility, including in the context of chronic childhood conditions, which can lead to overload, resilience difficulties, and changes in women’s quality of life^(21)^.

Furthermore, the meanings of Hope were constructed from facing the rehabilitation process, and from the desire to see the children fully rehabilitated in the future. Hope, for these caregivers, was also considered the one to give meaning to life, necessary to face the exhaustion of a long and challenging treatment. A study conducted in a municipality in southeastern Brazil reveals that caregivers believe hope is a resource that anticipates the future in relation to the child’s expectation to have health improved^(20)^.

It is also noteworthy that caregivers have dreams and expectations regarding the children’s future, as a form of resistance to the stigma they frequently experience. In these contexts, Hope is an important tool for strengthening children’s confidence in the future. A study conducted by Brazilian researchers identified that parents of children with orofacial clefts face emotional challenges, such as fear and sadness, from the moment the child is diagnosed, and these challenges are exacerbated by concerns about societal stigma^(22)^.

Thus, in line with what is proposed by SI, it is understood that Hope, both for the child and for the family caregiver, is built in social relationships, in affective interactions, and in the meanings attributed to the experiences lived in the rehabilitation process^(11)^.

It is noticeable that the toy has been reinterpreted as a safety factor and something positive. These results reinforce the need and importance of including play-based strategies in childcare, not just as a technique, but as a care tool capable of promoting hope and emotional health in children. Play therapy offers a safe space for emotional exploration, facilitating the child’s well-being and development^(23)^.

Although only the child’s primary family caregiver was included to deepen the understanding of Hope from the perspective of someone who experiences the rehabilitation process daily, this factor becomes a limitation, whereas including other family members would reinforce a broader understanding of Hope. Furthermore, it is suggested that other studies be carried out with children with orofacial clefts in broader age ranges, for example adolescents, which will allow us to understand other meanings of Hope.

Nevertheless, the study yields crucial results for improving care for children with cleft lip and palate and their families. The emphasis is placed on play-based strategies, such as therapeutic toys, which are a significant resource in rehabilitation contexts. In addition, the study demonstrates the importance of a holistic approach to this population, including Hope as a health need.

## CONCLUSION

The study showed that Hope is a highly relevant element in the care of children with orofacial clefts and for their family caregivers, being an important resource for coping with the rehabilitation process. For the children, Hope was attributed to emotional bonds, as well as to the transformation of self-image and professional future. For the caregivers, Hope was an inner strength sustained through spirituality and the desire to see the children rehabilitated.

Therefore, it is suggested that healthcare professionals integrate hope as a care need, recognizing its role in the rehabilitation process of children and, consequently, of their caregivers. Furthermore, integrating play-based techniques, such as therapeutic toys, with this population, can foster a welcoming and hopeful environment during the rehabilitation process. For family caregivers, it becomes necessary to incorporate psychosocial support actions that strengthen their coping resources.

## DATA AVAILABILITY

All the data supporting the results of this study were published in the article itself.
