# Supplementary material for: Senses and meanings attributed to Hope by children with orofacial clefts and their family caregivers
Source: Rev Esc Enferm USP. 2026 Aug 3;60:e20250561. doi: 10.1590/1980-220X-REEUSP-2025-0561en (PMC13450913; doi:10.1590/1980-220X-REEUSP-2025-0561en)
Supplement: Translation

# Translation: Sentidos e significados atribuídos à Esperança por crianças com fissura orofacial e seus cuidadores familiares*

**DOI:** 10.1590/1980-220X-REEUSP-2025-0561pt

**Authors:** Mariana Martire Mori, Camila Moraes Garollo Piran, Alana Vitória Escritori Cargnin, Armandos dos Santos Trettene, Bianca Machado Cruz Shibukawa, Zaida Borges Charepe, Marcela Demitto Furtado

**Affiliations:** 1 Universidade Estadual de Maringá, Programa de Pós-Graduação em Enfermagem, Maringá, PR, Brasil.; 2 Faculdade de Medicina de Bauru, Hospital de Reabilitação de Anomalias Craniofaciais, Bauru, SP, Brasil.; 3 Universidade Federal do Mato Grosso do Sul, Departamento de Enfermagem, Três Lagoas, MS, Brasil.; 4 Universidade Católica Portuguesa, Centro de Investigação Interdisciplinar em Saúde, Lisboa, Portugal.

**Keywords:** Criança, Cuidadores, Esperança, Fenda Labial, Fissura Palatina

## Abstract

**Objetivo:**

Compreender os sentidos e significados da Esperança para crianças com fissura orofacial e seus cuidadores familiares.

**Método:**

Estudo qualitativo, descritivo e exploratório, fundamentado no Modelo de Intervenção em Ajuda Mútua Promotor da Esperança e no Interacionismo Simbólico. Foi realizado em uma associação no Paraná, com crianças de cinco a dez anos com fissura orofacial e com cuidadores familiares de crianças menores de dez anos com a mesma condição. Os dados foram coletados por meio do Brinquedo Terapêutico Dramático e entrevistas semiestruturadas. Empregou-se a análise temática do tipo dedutiva, com apoio do *Atlas.ti* ^®^.

**Resultados:**

Participaram 16 crianças com fissura orofacial e 26 cuidadores familiares. Para as crianças, a Esperança foi atribuída à expectativa de coisas boas, incluindo desejos profissionais e transformação da autoimagem. Para os cuidadores, a Esperança foi associada à força interior e à espiritualidade, essencial para dar sentido à vida.

**Conclusão:**

A Esperança é uma dimensão essencial para crianças com fissura orofacial e seus cuidadores familiares no enfrentamento da reabilitação, sendo fundamental no processo de cuidado.

## INTRODUÇÃO

A Esperança é um fator essencial na promoção da saúde mental, capaz de proteger os indivíduos de sofrimento psíquico, promover o conforto e melhorar a qualidade de vida^(1)^. Trata-se de uma dimensão importante em contextos de vulnerabilidade, especialmente relacionados a problemas de saúde^(2)^. Ademais, é uma força dinâmica importante na promoção, manutenção e sustentação da vida, capaz de proteger as pessoas da ansiedade e do sofrimento^(3)^.

Refere-se a um recurso dinâmico construído por meio das relações sociais e vínculo entre os membros da família, e, no contexto de doenças crônicas pediátricas, a Esperança pode ser influenciada de acordo com o apoio social, o acesso a informações e a evolução no quadro de saúde^(4)^. Assim, em contextos de condições crônicas marcadas por incertezas e período prolongado de tratamento, a Esperança pode ser fortalecida ou fragilizada mediante a vivência das crianças e de seus cuidadores^(3)^. Na saúde infantil, a Esperança é um recurso que influencia de forma positiva o enfrentamento das adversidades, manifestada pelo estabelecimento de objetivos que as crianças buscam alcançar, a comportamentos positivos que contribuam no bem-estar e nas interações sociais ou crenças individuais^(5)^.

A fissura orofacial constitui uma das malformações congênitas mais frequentes, acometendo aproximadamente um a cada 650 nascidos vivos^(6)^. Trata-se de uma condição decorrente de alterações no desenvolvimento facial, cuja gravidade é variável e pode comprometer funções essenciais, como a alimentação, a fala e o desenvolvimento social da criança^(7)^. Os desafios psicológicos enfrentados por crianças com esta malformação são vários, principalmente relacionados à aparência e à autoestima, associados ao meio social e familiar em que estão inseridas^(8)^. Considerando o valor simbólico da aparência para as interações sociais, os estigmas e inseguranças quanto à aparência das crianças podem interferir na construção de expectativas positivas em relação ao futuro^(9)^.

Além de comprometer a saúde das crianças, as fissuras orofaciais têm potencial de alterar o bem-estar emocional dos seus cuidadores familiares, quando estes compartilham as vivências das crianças e oferecerem suporte e amparo ao longo do processo de reabilitação, marcado por procedimentos cirúrgicos, reabilitação da fala, atenção psicológica e saúde bucal^(10)^.

Mulheres responsáveis por crianças com fissura palatina frequentemente vivenciam intensos sentimentos de tristeza^(7)^. Essa experiência emocional pode ser explicada por diversos elementos, entre eles as intervenções clínicas, as exigências constantes do cuidado de um filho com demandas específicas, o julgamento social relacionado à aparência da criança, a escassa informação disponível à população em geral sobre a condição, bem como a limitada oferta de redes de apoio e assistência social^(10)^.

Dessa forma, de acordo com o Modelo de Intervenção em Ajuda Mútua Promotor da Esperança (MIAMPE), o apoio e a ajuda mútua são fatores que promovem a saúde mental e a Esperança de pessoas que vivenciam alguma condição crônica^(3)^. Somado a isso, o Interacionismo Simbólico (IS) revela que as interações sociais são relevantes para a construção compartilhada de significados entre os indivíduos^(11)^. Nessa perspectiva, torna-se fundamental considerar a capacidade da criança de construir sentidos sobre a Esperança e a fissura orofacial. Por conseguinte, abordar a Esperança para crianças com fissura orofacial e seus cuidadores familiares é uma forma de compreender a complexidade dos sentidos e significados de Esperança, proporcionando maior confiabilidade nos achados.

Nesse sentido, considerando que a fissura orofacial é uma condição crônica que possui um processo árduo de reabilitação, é necessário compreender o significado e o papel da Esperança para crianças com esta malformação e seus cuidadores familiares, a fim de incluir no plano de tratamento, além das condições clínicas, os fatores emocionais e sociais^(12)^. Ao integrar a Esperança ao planejamento terapêutico, o cuidado é ampliado para além de intervenções biomédicas, reconhecendo-a como um recurso de apoio no processo de reabilitação e um forte indicador de bem-estar emocional. Ademais, destaca-se a carência de estudos que abordam a Esperança com crianças com fissura orofacial e seus cuidadores, apontando uma lacuna de conhecimento. Assim, questiona-se: Quais os sentidos e significados de Esperança para crianças com fissura orofacial e seus cuidadores familiares? Portanto, este estudo tem como objetivo compreender os sentidos e significados da Esperança para crianças com fissura orofacial e seus cuidadores familiares.

## MÉTODO

### Tipo do Estudo

Estudo qualitativo, descritivo e exploratório ancorado no MIAMPE, o qual considera a Esperança em toda a sua amplitude, essencial para a promoção, manutenção e sustentação da vida, e proteção contra o sofrimento^(3)^. Também foi fundamentado no IS, teoria que considera que os símbolos, os significados e as interações sociais são importantes para a construção da realidade social, que pode ser moldada de acordo com a experiência^(11)^.

A escolha do MIAMPE justifica-se por considerar a Esperança em sua toda dimensão, como força essencial ao enfrentamento do sofrimento, especialmente em contextos de vulnerabilidade^(3)^. Já o IS permite compreender como os significados da Esperança são construídos nas interações sociais e mediados por símbolos^(11)^, fundamental para investigações subjetivas.

Foram seguidas as recomendações do guia *Consolidated criteria for reporting qualitative research (COREQ)*, a fim de garantir maior transparência ao estudo^(13)^.

### Local

O estudo foi realizado em uma associação de apoio que oferece atendimento a crianças e adolescentes com fissuras orofaciais e suas famílias, localizada no noroeste do estado do Paraná, Brasil, a qual tem como objetivo a reabilitação deste público. O atendimento oferecido na instituição é gratuito, vinculado ao Sistema Único de Saúde (SUS), e direcionado a 80 municípios, atendendo aproximadamente 500 pessoas e suas famílias.

### Participantes e Critérios de Seleção

O tamanho da amostra foi definido por conveniência, em que foram convidados a participar do estudo crianças com fissura orofacial, entre cinco e dez anos incompletos, e os cuidadores familiares de crianças menores de dez anos de idade com fissura orofacial, que compareceram para os atendimentos no serviço entre novembro de 2024 e abril de 2025. Entre os convidados a participar, não houve recusa. O recorte etário para as crianças foi escolhido devido ao período em que as crianças possuem a capacidade de representar a realidade que vivenciam, marcado pela brincadeira simbólica^(14)^. Por outro lado, foram considerados os cuidadores familiares de crianças menores de dez anos por apresentarem diferentes vivências, considerando a fase de reabilitação da criança, o que permitiria compreender a Esperança de uma forma mais abrangente.

Para as crianças, foram adotados como critérios de elegibilidade: crianças com fissura orofacial isolada (não associada a outras síndromes), dentro da faixa etária estabelecida e que frequentam o serviço com regularidade, conforme os agendamentos estabelecidos pela equipe. Quanto aos cuidadores familiares, foram considerados como critérios: ser o principal cuidador familiar e residir no mesmo domicílio da criança, considerando que a convivência diária possibilita o acompanhamento da rotina de reabilitação e favorece uma compreensão mais aprofundada dos fatores que influenciam a construção da Esperança nesse processo; participar de forma ativa no tratamento; e ter mais de 18 anos de idade. Para ambos, não foram incluídos aqueles que apresentassem déficit cognitivo atestado pelo médico, que influenciasse na compreensão das perguntas. Destaca-se que foram abordados os cuidadores familiares e as crianças de diversos núcleos familiares.

### Coleta de Dados

Os dados foram coletados entre novembro de 2024 e abril de 2025, pela pesquisadora principal, enfermeira e mestranda, que possui experiência com a técnica de coleta. A pesquisadora participa de um projeto de pesquisa na instituição há cinco anos e conhece a rotina do serviço; no entanto, não presta assistência direta às crianças e seus familiares. Foi realizado o contato com a diretora da instituição para verificar a melhor forma de conduzir a pesquisa, a fim de não atrapalhar a rotina de atendimentos. Destaca-se que os participantes foram selecionados a partir dos agendamentos das consultas, respeitando os critérios de elegibilidade determinados.

A coleta ocorreu na própria associação, em uma sala reservada e em ambiente calmo, sem interrupções, no intervalo entre as consultas, de forma individual. As crianças foram abordadas uma única vez cada, em uma sessão de Brinquedo Terapêutico Dramático (BTD) norteada pela pergunta: “Vamos brincar de uma criança que tem fissura orofacial e busca a Esperança?”. O BTD é uma estratégia que facilita o vínculo entre a criança e o pesquisador, e facilita a expressão verbal da criança^(14)^.

Foram oferecidos materiais diversos, incluindo bonecos para representar a família, profissionais de saúde, animais, brinquedos de uso doméstico e hospitalar, super-heróis e princesas, adesivos de emoções, bonecos com fissura labiopalatina, carrinhos, ônibus, massinhas de modelar e objetos simbólicos relacionados à espiritualidade (terço, cruz, anjo, vela, bíblia e imagem de Menino Jesus), os quais foram selecionados, devido ao reconhecimento sociocultural, como símbolos frequentemente associados a expressões de fé. Foi permitido que as crianças escolhessem aqueles com os quais mais se sentissem representadas (Figura 1). Os brinquedos foram recolhidos ao final da sessão. Cada sessão durou 50 minutos e foi registrada por meio de gravação audiovisual (vídeo e áudio), sob autorização dos responsáveis.

**Figura 1:** Brinquedos utilizados na pesquisa.

Fonte: Autores, 2025.

Para os cuidadores familiares, foi utilizada uma entrevista semiestruturada baseada no MIAMPE, guiada pela pergunta “O que é Esperança para você?”, e outras questões auxiliares. As entrevistas ocorreram uma única vez com cada cuidador, foram gravadas, com duração aproximada de 20 minutos cada, e, ao término, foi realizada a validação das falas por meio de um resumo oral apresentado ao participante, sendo permitido a ele retirar ou acrescentar algum conteúdo.

Os instrumentos de coleta foram validados, de forma simplificada, por três juízes doutores em enfermagem, que possuem afinidade com a área de saúde da criança, adolescente e famílias. Foi realizado um teste piloto com quatro crianças e quatro cuidadores, e, não se observando a necessidade de alterar os instrumentos, estas entrevistas foram incluídas na amostra final. As coletas foram finalizadas quando a pesquisadora observou que os dados já respondiam ao objetivo da pesquisa, por meio da análise e profundidade das falas^(15)^.

### Análise de Dados

As entrevistas foram transcritas integralmente à medida que eram realizadas. O material foi importado para o *software Atlas.ti* ^®^, uma ferramenta para auxiliar na organização, codificação e sistematização dos dados. A análise ocorreu de acordo com os pressupostos da análise temática dedutiva, seguindo as etapas: I-o material foi transcrito e lido de forma aprofundada; II-códigos foram criados de maneira sistemática; III-os temas foram buscados por meio dos códigos identificados; IV-a revisão dos temas foi realizada e um mapa temático criado no *software*; V-os temas foram nomeados; VI-as falas foram analisadas novamente, para verificar se correspondiam à pergunta de pesquisa^(16)^.

Visando ampliar a compreensão da Esperança e validar os dados, foi realizada a triangulação de dados a partir da coleta de informações com dois grupos, as crianças com fissura orofacial e os cuidadores familiares, o que permitiu contrastar os achados e enriquecer a análise^(17)^.

O processo passou por validação de três doutoras em enfermagem, em que, após a codificação inicial realizada pela pesquisadora principal, foram compartilhados os códigos e os temas acompanhados das falas, para garantir que o resultado retratasse com fidedignidade as falas dos participantes. A análise foi discutida em reuniões, em que foram examinadas as convergências e divergências na interpretação dos dados. Nos casos de discrepância, procedeu-se à revisão do material, em conjunto, até o consenso entre as pesquisadoras.

### Aspectos Éticos

O estudo foi realizado respeitando as diretrizes da Resolução nº 466/12 do Conselho Nacional de Saúde/Ministério da Saúde, e foi aprovado pelo Comitê de Ética em Pesquisa com Seres Humanos da universidade, sob parecer nº 7.041.250/2024.

A pesquisa foi explicada aos participantes, com uma linguagem acessível. Foi aplicado o Termo de Consentimento Livre e Esclarecido junto aos cuidadores familiares. Para as crianças, foi aplicado o Termo de Assentimento Livre e Esclarecido, o qual foi adaptado a uma linguagem acessível à faixa etária, incluindo elementos gráficos que facilitassem a compreensão.

Para garantir a preservação da identidade dos participantes, as crianças foram nomeadas de acordo com a inicial “C” de criança, seguida da ordem das entrevistas e a idade, enquanto os cuidadores familiares foram identificados de acordo com o grau de parentesco com a criança, seguido da ordem das entrevistas.

## RESULTADOS

Ao todo, 16 crianças participaram da pesquisa. Não houve predominância entre os sexos, sendo oito meninas e oito meninos; a idade variou de cinco a nove anos, e em relação ao tipo de fissura, oito eram pré-forame e oito, transforame.

Em relação aos cuidadores, 26 participaram, sendo 19 mães, dois pais, duas tias e três avós. A idade variou de 20 a 75 anos, sendo 92% (n = 24) do sexo feminino, 65% (n = 17) casados.

Observou-se que a Esperança possui significados diferentes para as crianças com fissura orofacial e os seus cuidadores familiares, e podem ser compreendidos por meio da Figura 2. Em ambos os grupos, a Esperança esteve relacionada ao futuro; no entanto, para as crianças, o futuro esteve relacionado ao futuro profissional. Por outro lado, para os cuidadores, o futuro é visto como a realização dos sonhos e a crença nas possibilidades da criança. A transformação da autoimagem foi relatada de forma significativa para as crianças. Para os cuidadores familiares, a Esperança esteve mais relacionada a uma força interior baseada na fé e espiritualidade.

**Figura 2:** Sentidos de Esperança para crianças com fissura orofacial e seus cuidadores familiares.

Fonte: Dados da pesquisa, 2025.

A partir da análise dos dados emergiram duas categorias temáticas: 1. Sentidos atribuídos à Esperança por crianças com fissura orofacial, e 2. Significados de Esperança para cuidadores familiares de crianças com fissuras orofaciais.

### 1. Sentidos Atribuídos à Esperança por Crianças com Fissura Orofacial

Os sentidos atribuídos à Esperança pelas crianças com fissura orofacial foram levantados utilizando a mediação do brinquedo. A Esperança foi relacionada a expectativa de coisas boas, superação da tristeza e a confiança mesmo diante das adversidades.

*[aponta para o ônibus, hulk e homem-aranha] São brinquedos, uma coisa boa. Esperança é quando fica boa.* (C7, 06 anos)

*A esperança é a última que morre. Você não pode perder a esperança enquanto você tem a sua vida. Quase tudo me dá esperança, bem quase, muito quase tudo me dá esperança.* (C12, 09 anos)

*[brincando de dentista] Que aconteçam coisas boas.* (C13, 08 anos)

*De encontrar uma coisa que está perdida e também que você tá muito triste e quer encontrar alguma coisa, quer fazer alguma coisa.* (C16, 07 anos)

As crianças atribuíram à Esperança com o desejo de transformação da autoimagem, e a projeção de futuros pais de crianças com fissura, reproduzindo os cuidados que receberam. Além disso, atribuem um significado negativo à vida adulta, devido ao preconceito da sociedade.

*Quando eu ficar grandona eu quero que a pele da minha boca saia, eu não gosto de ficar assim.* (C7, 06 anos)

*Antes de ir no trabalho passar maquiagem para ficar toda lisinha e não aparecer isso aqui [cicatriz]. Vai que os adultos veem e tiram sarro, porque eu não vou ser mais criança pras pessoas não tirarem sarro. Eu imagino que no futuro meus filhos vão ser tudo com a boquinha assim, eu só quero que aconteça com uma menina que vai ser minha filha porque eu vou achar fofinha [pega uma boneca para representar]. Se eu achar que ela não gostar eu vou falar que eu também tive. A minha filha, quando eu tiver, vai que ela não gosta, eu vou falar não tem problema você nasceu assim mesmo. Se ela se sentir mal eu vou levar ela no hospital e vou colocar ela aqui [associação].* (C16, 07 anos)

Durante a brincadeira, as crianças com fissura orofacial também expressaram Esperança relacionada ao futuro profissional, atribuindo significado aos profissionais responsáveis pela reabilitação, desejando exercer a mesma função no futuro. Os sentidos emergiram por meio dos brinquedos quando as crianças selecionaram bonecos e instrumentos médicos. Esses objetos foram utilizados para representar cenas cotidianas vivenciadas na reabilitação.

*Eu tô brincando de médico, a minha mãe me inspirou eu ser médica, porque ela me deu um brinquedo que era de médico, que tinha barbie, e daí eu ficava fazendo esses negócios de barbie, daí eu me inspirei a ser médica.* (C1, 07 anos)

*Eu quero ser professora, porque eu posso ajudar meus alunos e eu acho uma função boa. Eu queria ser médica, mas eu não consigo ver sangue e pessoa machucada. Eu acho uma função boa, porque várias pessoas podem se curar e não vão ficar doente.* (C11, 08 anos)

*Eu espero pelo menos ser alguém na vida, porque se Deus quiser eu vou fazer faculdade. Minha mãe fala para eu ir vendo já o que vou fazer. Mas todo mundo fala para eu fazer faculdade de medicina, mas não gosto muito, eu acho.* (C12, 09 anos)

A Esperança, para crianças com fissura orofacial, foi atribuída à expectativa de acontecer coisas boas e ao apoio para superar a tristeza, sendo permeada pelo desejo de transformação. Além disso, também esteve relacionada a confiança no futuro, relacionado ao tratamento e a realização profissional, muitas vezes inspirados nos profissionais que cuidam da própria reabilitação. Ademais, as crianças desejam reproduzir, no futuro, as situações que vivenciam durante a reabilitação, como o apoio e o cuidado, o que ressalta a importância dos bons exemplos.

### 2. Significados de Esperança para Cuidadores Familiares de Crianças com Fissuras Orofaciais

Para os cuidadores familiares de crianças com fissura, a Esperança remete ao significado da força interior, baseada na fé e na espiritualidade. Ainda, a Esperança está relacionada a coisas que acreditam, mas não necessariamente visualizam.

*Esperança eu acho que é que se a gente confia a gente consegue realizar através da nossa fé, não por aquilo que a gente vê, mas que a gente acredita.* (Mãe 05)

*Esperança é o que faz a gente acordar todo dia, de ter uma vida melhor, de conseguir ver os filhos crescerem.* (Mãe 06)

*É saber que alguma coisa vai dar certo mesmo que tudo esteja indo ao contrário.* (Mãe 16)

Os cuidadores associam a Esperança ao que fornece sentido durante a vida e o tratamento das crianças, acreditando que terão dias melhores. Os cuidadores consideram que a Esperança é essencial para preencher o vazio interior.

*A gente tem que ter esperança em alguma coisa na vida, a gente tem que ter alguma coisa para lutar por aquilo. Ter esperança me motiva mais ainda por acreditar, me faz ter mais vontade de procurar.* (Tia 02)

*A esperança é o que nos move, se a gente tem esperança a gente consegue ter uma vida e um futuro melhor. Acredito que uma pessoa sem esperança é uma pessoa vazia, porque não tem nada que preenche o vazio. Esperança é um sentimento, não é algo que a gente vê, concreto.* (Mãe 05)

*Eu acho que essa caminhada é muito difícil, e se a gente não tiver esperança a gente não sair do lugar.* (Mãe 15)

Alguns familiares atribuem Esperança ao futuro das crianças, reconhecendo as potencialidades e fornecendo o encorajamento. Para eles, a Esperança é um dia verem as crianças reabilitadas e realizando os sonhos.

*Eu falo pra ele assim, ‘o que você quer ser?’. A gente enche ele de esperança porque ele se achava não capaz, ele falava eu sou burro. Eu falo ‘o que você quer ser?’, ele fala que quer ser policial.* (Tia 02)

*A minha esperança pro futuro dela é ver minha filha bem reabilitada, gritando e falando dessa fissura que é a história dela, ela mostrar pra todo mundo que uma criança com fissura pode chegar aonde ela quiser, pode ser quem ela quiser.* (Mãe 03)

*A gente pensa assim, eu quero que você seja alguém, o que você quiser ser você é capaz de ser, não tem limite, não tem fronteira, vocês têm capacidade e conseguem.* (Mãe 05)

Para os cuidadores familiares, a Esperança é necessária para enfrentar a reabilitação, apoiada na fé e na espiritualidade. Além disso, oferece sentido à vida e proporciona a confiança na completa reabilitação das crianças e na realização dos sonhos.

## DISCUSSÃO

O sentido e o significado atribuídos à Esperança para crianças com fissura orofacial e seus cuidadores familiares são dinâmicos, projetados para o futuro, relacionados às interações sociais, aos vínculos afetivos, ao cuidado, à espiritualidade e ao processo de reabilitação. A Esperança assume funções diferentes para as crianças e seus cuidadores familiares. Os resultados da pesquisa apontam que, embora compartilhada, a Esperança assume funções distintas. Para as crianças, esteve relacionada a construção de identidade, enquanto para os cuidadores, é um recurso de sustentação emocional.

Para as crianças, a Esperança repercute no bem-estar, no apoio emocional e na superação da tristeza que pode ocorrer em momentos do processo de reabilitação. Estes resultados vão ao encontro dos pressupostos do MIAMPE, ao defender que a Esperança é uma força vital que ameniza o sofrimento e impulsiona as pessoas na busca por transformação^(3)^. De forma semelhante, um estudo desenvolvido com estudantes da China evidenciou que a Esperança está relacionada a uma boa saúde mental, por meio do fortalecimento da resiliência psicológica em contextos desafiadores^(18)^.

Por meio da brincadeira, as crianças representaram o desejo de um futuro profissional e de desempenharem profissões importantes no seu processo de reabilitação, como médicos, enfermeiros e dentistas, ressignificando o cuidado que recebem desses profissionais. Os achados reforçam como os significados são construídos socialmente a partir das experiências vividas^(11)^.

No entanto, contrapondo os achados deste estudo, uma pesquisa utilizando o desenho, realizada com crianças de uma escola brasileira, apontou que a profissão de cirurgião-dentista é vista pelas crianças de forma negativa, associada a expressões de medo e dor, e traços que remetem a tensão, ansiedade e emoções negativas^(19)^. Contudo, a vivência em procedimentos odontológicos é diferente entre essas crianças, visto que as crianças com fissura orofacial visualizam a profissão como uma possibilidade de melhora da imagem e das alterações causadas pela malformação.

As crianças também revelaram problemas com a autoimagem e a preocupação com o preconceito no futuro, na vida adulta. Dessa forma, consideram que a Esperança é fundamental para enfrentar o medo e a insegurança vivenciados no processo de reabilitação. Essa função também foi demonstrada em outros contextos de saúde, como é o caso de pacientes com acidente vascular cerebral, em que a Esperança funciona como um elemento que influencia de forma positiva a resiliência^(2)^.

Para os cuidadores familiares das crianças com fissura, a Esperança emergiu como uma força interior, que confere sentido à vida, sustentada pela espiritualidade. Tais percepções vão de encontro aos pressupostos do MIAMPE, em que a espiritualidade é um pilar que sustenta a Esperança diante de situações de adversidade^(3)^. Estes resultados são reforçados por um estudo realizado com cuidadores de crianças com condições crônicas diversas, que identificou que a Esperança atrelada a espiritualidade é um fator motivacional em suas vidas^(20)^.

A presente pesquisa apontou a predominância de mulheres, especialmente de mães, como principais cuidadores das crianças, evidenciando a centralidade feminina. Histórica e culturalmente, os cuidados são atribuídos como responsabilidade feminina, inclusive no contexto das condições crônicas infantis, o que pode causar sobrecarga, dificuldades de resiliência e alteração na qualidade de vida da mulher^(21)^.

Ademais, os significados de Esperança foram construídos a partir do enfrentamento do processo de reabilitação, e no desejo de ver as crianças completamente reabilitadas no futuro. A Esperança, para esses cuidadores, também foi considerada como aquilo que dá sentido à vida, necessária para enfrentar o cansaço do tratamento longo e desafiador. Estudo realizado em um município do Sudeste do Brasil revela que os cuidadores assumem que a Esperança é um recurso que antecipa o futuro em relação à expectativa de a criança obter melhoras no quadro de saúde^(20)^.

Destaca-se também que os cuidadores possuem sonhos e expectativas em relação ao futuro das crianças, como uma forma de resistência ao estigma frequentemente vivenciado. A Esperança, nesses contextos, é uma ferramenta importante para reforçar a confiança das crianças em relação ao futuro. Estudo realizado por pesquisadores brasileiros identificou que os pais de crianças com fissuras orofaciais enfrentam desafios emocionais, como o medo e a tristeza, desde o diagnóstico da criança, sentimentos que são potencializados pela preocupação com o estigma da sociedade^(22)^.

Assim, em consonância com o que é proposto pelo IS, compreende-se que a Esperança, tanto para a criança quanto para o cuidador familiar, é construída nas relações sociais, nas interações afetivas e nos significados atribuídos às experiências vividas no processo de reabilitação^(11)^.

Nota-se que o brinquedo foi ressignificado como um fator de segurança e algo positivo. Esses resultados reforçam a necessidade e a importância de incluir estratégias lúdicas na assistência às crianças, não apenas como uma técnica, mas uma ferramenta de cuidado capaz de promover a Esperança e a saúde emocional das crianças. A terapia por meio do brincar oferece um espaço seguro para a exploração emocional, facilitando o bem-estar e o desenvolvimento da criança^(23)^.

Ainda que tenham sido incluídos apenas o principal cuidador familiar da criança visando aprofundar o entendimento da Esperança a partir da perspectiva daquele que vivencia cotidianamente o processo de reabilitação, este fator torna-se uma limitação, ao passo que a inclusão dos demais membros da família reforçariam a compreensão mais ampla de Esperança. Além disso, sugere-se a realização de outros estudos com crianças com fissura orofacial em faixas etárias mais amplas, por exemplo adolescentes, o que permitirá compreender outros sentidos de Esperança.

Ainda assim, o estudo traz resultados fundamentais para a melhoria da assistência a crianças com fissura orofacial e seus familiares. Destaca-se a valorização de estratégias lúdicas, como o brinquedo terapêutico, um recurso significativo nos contextos de reabilitação. Somado a isso, o estudo demonstra a importância de uma abordagem integral a este público, incluindo a Esperança como uma necessidade de saúde.

## CONCLUSÃO

O estudo evidenciou que a Esperança é um elemento de grande relevância no cuidado às crianças com fissura orofacial e seus cuidadores familiares, sendo um recurso importante para o enfrentamento do processo de reabilitação. Para as crianças, a Esperança foi atribuída aos vínculos afetivos, assim como na transformação da autoimagem e o futuro profissional. Para os cuidadores, a Esperança foi uma força interior sustentada pela espiritualidade e o desejo de verem as crianças reabilitadas.

Assim, sugere-se que os profissionais da saúde integrem a Esperança como uma necessidade de cuidado, reconhecendo o seu papel no processo de reabilitação das crianças e consequentemente de seus cuidadores. Além disso, integrar técnicas lúdicas, como o brinquedo terapêutico, com esse público, pode favorecer um ambiente acolhedor e promotor de Esperança durante o processo de reabilitação. Para os cuidadores familiares, torna-se necessário incorporar ações de apoio psicossocial que fortaleçam seus recursos de enfrentamento.

## DISPONIBILIDADE DE DADOS

Todo o conjunto de dados que dá suporte aos resultados deste estudo foi publicado no próprio artigo.

# Translation: Sentidos y significados atribuidos a la Esperanza por parte de niños con fisura orofacial y sus cuidadores familiares*

**DOI:** 10.1590/1980-220X-REEUSP-2025-0561es

**Authors:** Mariana Martire Mori, Camila Moraes Garollo Piran, Alana Vitória Escritori Cargnin, Armandos dos Santos Trettene, Bianca Machado Cruz Shibukawa, Zaida Borges Charepe, Marcela Demitto Furtado

**Affiliations:** 1 Universidade Estadual de Maringá, Programa de Pós-Graduação em Enfermagem, Maringá, PR, Brasil.; 2 Faculdade de Medicina de Bauru, Hospital de Reabilitação de Anomalias Craniofaciais, Bauru, SP, Brasil.; 3 Universidade Federal do Mato Grosso do Sul, Departamento de Enfermagem, Três Lagoas, MS, Brasil.; 4 Universidade Católica Portuguesa, Centro de Investigação Interdisciplinar em Saúde, Lisboa, Portugal.

**Keywords:** Niño, Cuidadores, Esperanza, Labio Leporino, Fisura del Paladar

## Abstract

**Objetivo:**

Comprender los sentidos y significados de la Esperanza para los niños con fisura orofacial y sus cuidadores familiares.

**Método:**

Estudio cualitativo, descriptivo y exploratorio, basado en el Modelo de Intervención en Ayuda Mutua Promotor de la Esperanza y en el Interaccionismo Simbólico. Se llevó a cabo en una asociación de Paraná, con niños de cinco a diez años con fisura orofacial y con cuidadores familiares de niños menores de diez años con la misma afección. Los datos se recopilaron mediante el Juego Terapéutico Dramático y entrevistas semiestructuradas. Se empleó el análisis temático de tipo deductivo, con el apoyo de *Atlas.ti* ^®^.

**Resultados:**

Participaron 16 niños con fisura orofacial y 26 cuidadores familiares. Para los niños, la Esperanza se atribuyó a la expectativa de cosas buenas, incluyendo deseos profesionales y la transformación de la autoimagen. Para los cuidadores, la Esperanza se asoció con la fuerza interior y la espiritualidad, esenciales para dar sentido a la vida.

**Conclusión:**

La Esperanza es una dimensión esencial para los niños con fisura orofacial y sus cuidadores familiares al enfrentar la rehabilitación, siendo fundamental en el proceso de cuidado.

## INTRODUCCIÓN

La Esperanza es un factor esencial en la promoción de la salud mental, capaz de proteger a las personas del sufrimiento psíquico, promover el bienestar y mejorar la calidad de vida^(1)^. Se trata de una dimensión importante en contextos de vulnerabilidad, especialmente relacionados con problemas de salud^(2)^. Además, es una fuerza dinámica importante en la promoción, el mantenimiento y el sostenimiento de la vida, capaz de proteger a las personas de la ansiedad y el sufrimiento^(3)^.

Se refiere a un recurso dinámico construido a través de las relaciones sociales y el vínculo entre los miembros de la familia, y, en el contexto de las enfermedades crónicas pediátricas, la Esperanza puede verse influida por el apoyo social, el acceso a la información y la evolución del cuadro de salud^(4)^. Así, en contextos de afecciones crónicas marcadas por la incertidumbre y un período prolongado de tratamiento, la Esperanza puede fortalecerse o debilitarse a través de las experiencias de los niños y sus cuidadores^(3)^. En la salud infantil, la Esperanza es un recurso que influye positivamente en la superación de las adversidades, manifestándose en el establecimiento de objetivos que los niños buscan alcanzar, en comportamientos positivos que contribuyen al bienestar y en las interacciones sociales o las creencias individuales^(5)^.

La fisura orofacial constituye una de las malformaciones congénitas más frecuentes, afectando aproximadamente a uno de cada 650 nacidos vivos^(6)^. Se trata de una afección derivada de alteraciones en el desarrollo facial, cuya gravedad es variable y puede comprometer funciones esenciales, como la alimentación, el habla y el desarrollo social del niño^(7)^. Los desafíos psicológicos que enfrentan los niños con esta malformación son diversos, principalmente relacionados con la apariencia y la autoestima, asociados al entorno social y familiar en el que se insertan^(8)^. Considerando el valor simbólico de la apariencia para las interacciones sociales, los estigmas y las inseguridades respecto a la apariencia de los niños pueden interferir en la construcción de expectativas positivas en relación con el futuro^(9)^.

Además de comprometer la salud de los niños, las fisuras orofaciales tienen el potencial de alterar el bienestar emocional de sus cuidadores familiares, cuando estos comparten las experiencias de los niños y les ofrecen apoyo y protección a lo largo del proceso de rehabilitación, marcado por procedimientos quirúrgicos, rehabilitación del habla, atención psicológica y salud bucal^(10)^.

Las mujeres a cargo de niños con fisura palatina suelen experimentar intensos sentimientos de tristeza^(7)^. Esta experiencia emocional puede explicarse por diversos elementos, entre ellos las intervenciones clínicas, las exigencias constantes del cuidado de un hijo con necesidades específicas, el juicio social relacionado con la apariencia del niño, la escasa información disponible para la población en general sobre la afección, así como la oferta limitada de redes de apoyo y asistencia social^(10)^.

De este modo, de acuerdo con el Modelo de Intervención en Ayuda Mutua Promotor de la Esperanza (MIAMPE), el apoyo y la ayuda mutua son factores que promueven la salud mental y la Esperanza de las personas que viven con alguna afección crónica^(3)^. Además, el Interaccionismo Simbólico (IS) revela que las interacciones sociales son relevantes para la construcción compartida de significados entre los individuos^(11)^. Desde esta perspectiva, resulta fundamental considerar la capacidad del niño para construir significados sobre la Esperanza y la fisura orofacial. Por consiguiente, abordar la Esperanza en el caso de los niños con fisura orofacial y sus cuidadores familiares es una forma de comprender la complejidad de los sentidos y significados de la Esperanza, lo que proporciona una mayor confiabilidad a los hallazgos.

En este sentido, considerando que la fisura orofacial es una afección crónica que conlleva un arduo proceso de rehabilitación, es necesario comprender el significado y el papel de la Esperanza para los niños con esta malformación y sus cuidadores familiares, a fin de incluir en el plan de tratamiento, además de las condiciones clínicas, los factores emocionales y sociales^(12)^. Al integrar la Esperanza en la planificación terapéutica, la atención se amplía más allá de las intervenciones biomédicas, reconociéndola como un recurso de apoyo en el proceso de rehabilitación y un fuerte indicador de bienestar emocional. Además, se destaca la falta de estudios que aborden la Esperanza en niños con fisura orofacial y sus cuidadores, lo que señala una laguna de conocimiento. Por lo tanto, se plantea la pregunta: ¿Cuáles son los sentidos y significados de la Esperanza para los niños con fisura orofacial y sus cuidadores familiares? Por lo tanto, este estudio tiene como objetivo comprender los sentidos y significados de la Esperanza para los niños con fisura orofacial y sus cuidadores familiares.

## MÉTODO

### Tipo de Estudio

Estudio cualitativo, descriptivo y exploratorio basado en el MIAMPE, que considera la Esperanza en toda su amplitud, esencial para la promoción, el mantenimiento y la sustentación de la vida, y la protección contra el sufrimiento^(3)^. También se basó en la IS, teoría que considera que los símbolos, los significados y las interacciones sociales son importantes para la construcción de la realidad social, que puede moldearse de acuerdo con la experiencia^(11)^.

La elección del MIAMPE se justifica por considerar la Esperanza en toda su dimensión, como fuerza esencial para enfrentar el sufrimiento, especialmente en contextos de vulnerabilidad^(3)^. Por su parte, la IS permite comprender cómo los significados de la Esperanza se construyen en las interacciones sociales y son mediados por símbolos^(11)^, lo cual es fundamental para las investigaciones subjetivas.

Se siguieron las recomendaciones de la guía *Consolidated criteria for reporting qualitative research (COREQ)*, con el fin de garantizar una mayor transparencia al estudio^(13)^.

### Lugar

El estudio se llevó a cabo en una asociación de apoyo que ofrece atención a niños y adolescentes con fisuras orofaciales y a sus familias, ubicada en el noroeste del estado de Paraná, Brasil, cuyo objetivo es la rehabilitación de este público. La atención ofrecida en la institución es gratuita, está vinculada al Sistema Único de Salud (SUS) y se dirige a 80 municipios, atendiendo a aproximadamente 500 personas y sus familias.

### Participantes y Criterios de Selección

El tamaño de la muestra se definió por conveniencia, invitándose a participar en el estudio a niños con fisura orofacial, de entre cinco y diez años no cumplidos, y a los cuidadores familiares de niños menores de diez años con fisura orofacial, que acudieron a las consultas del servicio entre noviembre de 2024 y abril de 2025. Entre los invitados a participar, no hubo rechazos. El rango de edad para los niños se eligió debido al período en el que los niños tienen la capacidad de representar la realidad que viven, marcado por el juego simbólico^(14)^. Por otro lado, se consideró a los cuidadores familiares de niños menores de diez años por presentar diferentes experiencias, teniendo en cuenta la fase de rehabilitación del niño, lo que permitiría comprender la Esperanza de una manera más amplia.

Para los niños, se adoptaron como criterios de elegibilidad: niños con fisura orofacial aislada (no asociada a otros síndromes), dentro del rango de edad establecido y que asisten al servicio con regularidad, de acuerdo con los horarios establecidos por el equipo. En cuanto a los cuidadores familiares, se consideraron como criterios: ser el cuidador familiar principal y residir en el mismo domicilio que el niño, considerando que la convivencia diaria permite el seguimiento de la rutina de rehabilitación y favorece una comprensión más profunda de los factores que influyen en la construcción de la Esperanza en este proceso; participar de manera activa en el tratamiento; y tener más de 18 años de edad. En ambos casos, no se incluyó a quienes presentaran un déficit cognitivo certificado por el médico que influyera en la comprensión de las preguntas. Cabe destacar que se abordó a cuidadores familiares y niños de diversos núcleos familiares.

### Recolección de Datos

Los datos fueron recolectados entre noviembre de 2024 y abril de 2025 por la investigadora principal, enfermera y estudiante de maestría, quien cuenta con experiencia en la técnica de recolección. La investigadora participa en un proyecto de investigación en la institución desde hace cinco años y conoce la rutina del servicio; sin embargo, no brinda atención directa a los niños ni a sus familiares. Se contactó a la directora de la institución para verificar la mejor manera de llevar a cabo la investigación, con el fin de no perturbar la rutina de la atención. Cabe destacar que los participantes fueron seleccionados a partir de las citas programadas, respetando los criterios de elegibilidad establecidos.

La recolección de datos se llevó a cabo en la propia asociación, en una sala reservada y en un ambiente tranquilo, sin interrupciones, en el intervalo entre consultas, de manera individual. Se abordó a los niños una sola vez a cada uno, en una sesión de Juguete Terapéutico Dramático (JTD) orientada por la pregunta: “¿Jugamos a ser un niño que tiene fisura orofacial y busca la Esperanza?”. El JTD es una estrategia que facilita el vínculo entre el niño y el investigador, y facilita la expresión verbal del niño^(14)^.

Se ofrecieron diversos materiales, incluyendo muñecos para representar a la amilia, profesionales de la salud, animales, juguetes de uso doméstico y hospitalario, superhéroes y princesas, pegatinas de emociones, muñecos con fisura labio-palatina, carritos, autobuses, plastilina y objetos simbólicos relacionados con la espiritualidad (rosario, cruz, ángel, vela, Biblia e imagen del Niño Jesús), los cuales fueron seleccionados, debido a su reconocimiento sociocultural, como símbolos frecuentemente asociados a expresiones de fe. Se permitió a los niños elegir aquellos con los que más se sintieran representados (Figura 1). Los juguetes se recogieron al final de la sesión. Cada sesión duró 50 minutos y se registró mediante grabación audiovisual (vídeo y audio), con la autorización de los responsables.

**Figura 1:** Juguetes utilizados en la investigación.

Fuente: Autores, 2025.

Para los cuidadores familiares, se utilizó una entrevista semiestructurada basada en el MIAMPE, guiada por la pregunta “¿Qué es la Esperanza para usted?”, y otras preguntas complementarias. Las entrevistas se realizaron una sola vez con cada cuidador, se grabaron, tuvieron una duración aproximada de 20 minutos cada una y, al finalizar, se llevó a cabo la validación de los comentarios mediante un resumen oral presentado al participante, permitiéndole retirar o agregar algún contenido.

Los instrumentos de recolección fueron validados, de manera simplificada, por tres jueces con doctorado en enfermería, que tienen afinidad con el área de salud de la infancia, la adolescencia y las familias. Se realizó una prueba piloto con cuatro niños y cuatro cuidadores, y, al no observarse la necesidad de modificar los instrumentos, estas entrevistas se incluyeron en la muestra final. La recolección de datos concluyó cuando la investigadora observó que los datos ya respondían al objetivo de la investigación, a través del análisis y la profundidad de los discursos^(15)^.

### Análisis de Datos

Las entrevistas se transcribieron íntegramente a medida que se realizaban. El material se importó al *software Atlas.ti* ^®^, una herramienta para ayudar en la organización, codificación y sistematización de los datos. El análisis se llevó a cabo de acuerdo con los supuestos del análisis temático deductivo, siguiendo las siguientes etapas: I-el material se transcribió y se leyó en profundidad; II-se crearon códigos de manera sistemática; III-se identificaron los temas a partir de los códigos; IV-se revisaron los temas y se creó un mapa temático en el *software*; V-se nombraron los temas; VI-se analizaron nuevamente los discursos para verificar si se correspondían con la pregunta de investigación^(16)^.

Con el fin de ampliar la comprensión de la Esperanza y validar los datos, se llevó a cabo la triangulación de datos a partir de la recolección de información con dos grupos: los niños con fisura orofacial y los cuidadores familiares, lo que permitió contrastar los hallazgos y enriquecer el análisis^(17)^.

El proceso fue validado por tres doctoradas en enfermería, en el que, tras la codificación inicial realizada por la investigadora principal, se compartieron los códigos y los temas acompañados de los discursos, para garantizar que el resultado reflejara fielmente los discursos de los participantes. El análisis se discutió en reuniones, en las que se examinaron las convergencias y divergencias en la interpretación de los datos. En los casos de discrepancia, se procedió a la revisión conjunta del material hasta alcanzar el consenso entre las investigadoras.

### Aspectos Éticos

El estudio se llevó a cabo respetando las directrices de la Resolución n.º 466/12 del Consejo Nacional de Salud/Ministerio de Salud, y fue aprobado por el Comité de Ética en Investigación con Seres Humanos de la universidad, bajo el dictamen n.º 7.041.250/2024.

La investigación se explicó a los participantes en un lenguaje accesible. Se aplicó el Término de Consentimiento Libre e Informado a los cuidadores familiares. Para los niños, se aplicó el Término de Asentimiento Libre e Informado, el cual se adaptó a un lenguaje accesible para el grupo de edad, incluyendo elementos gráficos que facilitaran la comprensión.

Para garantizar la preservación de la identidad de los participantes, a los niños se les asignó un nombre según la inicial “C” de niño (criança en portugués), seguida del orden de las entrevistas y la edad, mientras que a los cuidadores familiares se les identificó según el grado de parentesco con el niño, seguido del orden de las entrevistas.

## RESULTADOS

En total, participaron en la investigación 16 niños. No hubo predominio entre los sexos, siendo ocho niñas y ocho niños; la edad varió de cinco a nueve años, y en cuanto al tipo de fisura, ocho eran prefora y ocho, transfora.

En cuanto a los cuidadores, participaron 26, de los cuales 19 eran madres, dos padres, dos tías y tres abuelos. La edad osciló entre los 20 y los 75 años, siendo el 92 % (n = 24) mujeres y el 65 % (n = 17) casados.

Se observó que la Esperanza tiene significados diferentes para los niños con fisura orofacial y sus cuidadores familiares, y puede entenderse a través de la Figura 2. En ambos grupos, la Esperanza se relacionó con el futuro; sin embargo, para los niños, el futuro se relacionó con el futuro profesional. Por otro lado, para los cuidadores, el futuro se ve como la realización de los sueños y la creencia en las posibilidades del niño. La transformación de la autoimagen se reportó de manera significativa para los niños. Para los cuidadores familiares, la Esperanza se relacionó más con una fuerza interior basada en la fe y la espiritualidad.

**Figura 2:** Sentidos de la Esperanza para los niños con fisura orofacial y sus cuidadores familiares.

Fuente: Datos de la investigación, 2025.

A partir del análisis de los datos surgieron dos categorías temáticas: 1. Sentidos atribuidos a la Esperanza por los niños con fisura orofacial, y 2. Significados de la Esperanza para los cuidadores familiares de niños con fisuras orofaciales.

### 1. Sentidos Atribuidos a la Esperanza por los Niños con Fisura Orofacial

Los significados atribuidos a la Esperanza por los niños con fisura orofacial se recopilaron utilizando el juguete como mediador. La Esperanza se relacionó con la expectativa de cosas buenas, la superación de la tristeza y la confianza incluso ante las adversidades.

*[señala el autobús, a Hulk y a Spider-Man] Son juguetes, algo bueno. La Esperanza es cuando las cosas se ponen bien.*(C7, 6 años)

*La Esperanza es lo último que se pierde. No puedes perder la Esperanza mientras tengas vida. Casi todo me da Esperanza, bueno, casi, casi todo me da Esperanza.* (C12, 9 años)

*[jugando al dentista] Que pasen cosas buenas.* (C13, 8 años)

*Encontrar algo que se ha perdido y también cuando estás muy triste y quieres encontrar algo, quieres hacer algo.* (C16, 7 años)

Los niños asociaron la Esperanza con el deseo de transformar su autoimagen y con la proyección de futuros padres de niños con fisura labial, reproduciendo los cuidados que ellos mismos recibieron. Además, atribuyen un significado negativo a la vida adulta, debido al prejuicio de la sociedad.

*Cuando sea grande quiero que se me quite la piel de la boca, no me gusta estar así.* (C7, 6 años)

*Antes de ir al trabajo me maquillo para que quede todo liso y no se vea esto de aquí [cicatriz]. A ver si los adultos lo ven y se burlan, porque ya no voy a ser niña para que la gente no se burle. Me imagino que en el futuro mis hijos van a tener todos la boquita así, solo quiero que le pase a una niña que vaya a ser mi hija porque me va a parecer linda [toma una muñeca para representarla]. Si me parece que a ella no le gusta, le diré que a mí también me pasó. Mi hija, cuando la tenga, si no le gusta, le diré que no hay problema, que naciste así. Si se siente mal, la llevaré al hospital y la inscribiré aquí [asociación].* (C16, 7 años)

Durante el juego, los niños con fisura orofacial también expresaron Esperanza relacionada con su futuro profesional, atribuyendo importancia a los profesionales responsables de la rehabilitación y deseando ejercer la misma función en el futuro. Estos significados surgieron a través de los juguetes cuando los niños seleccionaron muñecos e instrumentos de salud. Estos objetos se utilizaron para representar escenas cotidianas vividas en la rehabilitación.

*Estoy jugando a ser doctora, mi mamá me inspiró a ser médica, porque me regaló un juguete de médicos, que tenía una Barbie, y entonces yo jugaba con la Barbie, y así me inspiré para ser doctora.* (C1, 7 años)

*Quiero ser maestra, porque puedo ayudar a mis alumnos y me parece un buen trabajo. Quería ser médica, pero no soporto ver sangre ni a personas heridas. Me parece un buen trabajo, porque muchas personas pueden curarse y no se van a enfermar.* (C11, 8 años)

*Espero al menos ser alguien en la vida, porque si Dios quiere voy a estudiar en la universidad. Mi mamá me dice que ya empiece a pensar en lo que voy a hacer. Pero todos me dicen que estudie medicina, pero no me gusta mucho, creo.* (C12, 9 años)

La Esperanza, para los niños con fisura orofacial, se atribuyó a la expectativa de que sucedan cosas buenas y al apoyo para superar la tristeza, impregnada por el deseo de transformación. Además, también se relacionó con la confianza en el futuro, en relación con el tratamiento y la realización profesional, a menudo inspirados en los profesionales que se ocupan de la propia rehabilitación. Por otra parte, los niños desean reproducir, en el futuro, las situaciones que viven durante la rehabilitación, como el apoyo y el cuidado, lo que resalta la importancia de los buenos ejemplos.

### 2. Significados de la Esperanza para los Cuidadores Familiares de Niños con Fisuras Orofaciales

Para los cuidadores familiares de niños con fisura, la Esperanza remite al significado de la fuerza interior, basada en la fe y la espiritualidad. Además, la Esperanza está relacionada con cosas en las que creen, pero que no necesariamente visualizan.

*Creo que la Esperanza es que, si confiamos, logramos alcanzar nuestras metas a través de nuestra fe, no por lo que vemos, sino por lo que creemos.* (Madre 05)

*La Esperanza es lo que nos hace despertarnos cada día, con la ilusión de tener una vida mejor, de poder ver crecer a nuestros hijos.* (Madre 06)

*Es saber que algo va a salir bien aunque todo vaya al revés.* (Madre 16)

Los cuidadores asocian la Esperanza con lo que da sentido a la vida y al cuidado de los niños, creyendo que tendrán días mejores. Los cuidadores consideran que la Esperanza es esencial para llenar el vacío interior.

*Tenemos que tener Esperanza en algo en la vida, tenemos que tener algo por lo que luchar. Tener Esperanza me motiva aún más a creer, me da más ganas de buscar.* (Tía 02)

*La Esperanza es lo que nos mueve; si tenemos Esperanza, podemos tener una vida y un futuro mejores. Creo que una persona sin Esperanza es una persona vacía, porque no tiene nada que llene ese vacío. La Esperanza es un sentimiento, no es algo que veamos, algo concreto.* (Mamá 05)

*Creo que este camino es muy difícil, y si no tenemos Esperanza, no saldremos del lugar.* (Mamá 15)

Algunos familiares atribuyen Esperanza al futuro de los niños, reconociendo su potencial y brindándoles aliento. Para ellos, la Esperanza es ver algún día a los niños rehabilitados y haciendo realidad sus sueños.

*Le digo así: “¿Qué quieres ser?”. Le llenamos de Esperanza porque él se sentía incapaz, decía que era tonto. Le digo: “¿Qué quieres ser?”, y él dice que quiere ser policía.* (Tía 02)

*Mi Esperanza para su futuro es ver a mi hija bien rehabilitada, gritando y contando esa historia tan increíble que es la suya, que le demuestre a todo el mundo que una niña con fisura puede llegar a donde quiera, puede ser quien quiera.*(Mamá 03)

*Pensamos así: quiero que seas alguien, lo que quieras ser, eres capaz de serlo, no hay límites, no hay fronteras, tienen capacidad y lo logran.* (Madre 05)

Para los cuidadores familiares, la Esperanza es necesaria para afrontar la rehabilitación, apoyada en la fe y la espiritualidad. Además, da sentido a la vida y proporciona confianza en la completa rehabilitación de los niños y en la realización de sus sueños.

## DISCUSIÓN

El sentido y el significado atribuidos a la Esperanza para los niños con fisura orofacial y sus cuidadores familiares son dinámicos, proyectados hacia el futuro, relacionados con las interacciones sociales, los vínculos afectivos, el cuidado, la espiritualidad y el proceso de rehabilitación. La Esperanza asume funciones diferentes para los niños y sus cuidadores familiares. Los resultados de la investigación indican que, aunque compartida, la Esperanza asume funciones distintas. Para los niños, se relacionó con la construcción de la identidad, mientras que para los cuidadores es un recurso de apoyo emocional.

Para los niños, la Esperanza repercute en el bienestar, el apoyo emocional y la superación de la tristeza que puede surgir en momentos del proceso de rehabilitación. Estos resultados concuerdan con los supuestos del MIAMPE, al defender que la Esperanza es una fuerza vital que alivia el sufrimiento e impulsa a las personas en la búsqueda de la transformación^(3)^. De manera similar, un estudio realizado con estudiantes de China evidenció que la Esperanza está relacionada con una buena salud mental, mediante el fortalecimiento de la resiliencia psicológica en contextos desafiantes^(18)^.

A través del juego, los niños representaron el deseo de un futuro profesional y de desempeñar profesiones importantes en su proceso de rehabilitación, como médicos, enfermeros y dentistas, re-significando el cuidado que reciben de estos profesionales. Los hallazgos refuerzan cómo los significados se construyen socialmente a partir de las experiencias vividas^(11)^.

Sin embargo, en contraposición a los hallazgos de este estudio, una investigación que utilizó el dibujo, realizada con niños de una escuela brasileña, señaló que la profesión de cirujano-dentista es vista por los niños de manera negativa, asociada a expresiones de miedo y dolor, y rasgos que remiten a la tensión, la ansiedad y las emociones negativas^(19)^. No obstante, la experiencia en procedimientos odontológicos es diferente entre estos niños, ya que los niños con fisura orofacial visualizan la profesión como una posibilidad de mejorar la imagen y las alteraciones causadas por la malformación.

Los niños también revelaron problemas con la autoimagen y la preocupación por el prejuicio en el futuro, en la vida adulta. De este modo, consideran que la Esperanza es fundamental para enfrentar el miedo y la inseguridad vividos en el proceso de rehabilitación. Esta función también se ha demostrado en otros contextos de salud, como es el caso de pacientes con accidente cerebrovascular, en los que la Esperanza funciona como un elemento que influye positivamente en la resiliencia^(2)^.

Para los cuidadores familiares de los niños con fisura, la Esperanza surgió como una fuerza interior que da sentido a la vida, sustentada por la espiritualidad. Tales percepciones concuerdan con los supuestos del MIAMPE, en los que la espiritualidad es un pilar que sustenta la Esperanza ante situaciones de adversidad^(3)^. Estos resultados se ven reforzados por un estudio realizado con cuidadores de niños con diversas afecciones crónicas, que identificó que la Esperanza vinculada a la espiritualidad es un factor motivacional en sus vidas^(20)^.

La presente investigación señaló el predominio de las mujeres, especialmente de las madres, como principales cuidadoras de los niños, lo que pone de manifiesto la centralidad femenina. Histórica y culturalmente, los cuidados se atribuyen como una responsabilidad femenina, incluso en el contexto de las afecciones crónicas infantiles, lo que puede causar sobrecarga, dificultades de resiliencia y alteraciones en la calidad de vida de la mujer^(21)^.

Además, los significados de la Esperanza se construyeron a partir del enfrentamiento al proceso de rehabilitación y del deseo de ver a los niños completamente rehabilitados en el futuro. La Esperanza, para estos cuidadores, también se consideró como aquello que da sentido a la vida, necesaria para enfrentar el cansancio del tratamiento largo y desafiante. Un estudio realizado en un municipio del sudeste de Brasil revela que los cuidadores asumen que la Esperanza es un recurso que anticipa el futuro en relación con la expectativa de que el niño mejore su estado de salud^(20)^.

También se destaca que los cuidadores tienen sueños y expectativas respecto al futuro de los niños, como una forma de resistencia al estigma que suelen experimentar. La Esperanza, en estos contextos, es una herramienta importante para reforzar la confianza de los niños en relación con el futuro. Un estudio realizado por investigadores brasileños identificó que los padres de niños con fisuras orofaciales enfrentan desafíos emocionales, como el miedo y la tristeza, desde el diagnóstico del niño, sentimientos que se ven potenciados por la preocupación por el estigma de la sociedad^(22)^.

Así, en consonancia con lo que propone el IS, se entiende que la Esperanza, tanto para la niña como para el cuidador familiar, se construye en las relaciones sociales, en las interacciones afectivas y en los significados atribuidos a las experiencias vividas en el proceso de rehabilitación^(11)^.

Se observa que el juguete ha adquirido un nuevo significado como factor de seguridad y algo positivo. Estos resultados refuerzan la necesidad y la importancia de incluir estrategias lúdicas en la atención a los niños, no solo como una técnica, sino como una herramienta de cuidado capaz de promover la Esperanza y la salud emocional de los niños. La terapia a través del juego ofrece un espacio seguro para la exploración emocional, facilitando el bienestar y el desarrollo del niño^(23)^.

Aunque solo se incluyó al cuidador familiar principal del niño con el fin de profundizar en la comprensión de la Esperanza desde la perspectiva de quien vive a diario el proceso de rehabilitación, este factor se convierte en una limitación, ya que la inclusión de los demás miembros de la familia reforzaría una comprensión más amplia de la Esperanza. Además, se sugiere la realización de otros estudios con niños con fisura orofacial en rangos de edad más amplios, por ejemplo, adolescentes, lo que permitirá comprender otros significados de la Esperanza.

Aun así, el estudio aporta resultados fundamentales para la mejora de la atención a los niños con fisura orofacial y a sus familiares. Cabe destacar la importancia de las estrategias lúdicas, como el juguete terapéutico, un recurso significativo en los contextos de rehabilitación. Además, el estudio demuestra la importancia de un enfoque integral para este público, incluyendo la Esperanza como una necesidad de salud.

## CONCLUSIÓN

El estudio evidenció que la Esperanza es un elemento de gran relevancia en el cuidado de los niños con fisura orofacial y sus cuidadores familiares, siendo un recurso importante para afrontar el proceso de rehabilitación. Para los niños, la Esperanza se atribuyó a los vínculos afectivos, así como a la transformación de la autoimagen y el futuro profesional. Para los cuidadores, la Esperanza fue una fuerza interior sustentada por la espiritualidad y el deseo de ver a los niños rehabilitados.

Por lo tanto, se sugiere que los profesionales de la salud integren la Esperanza como una necesidad de atención, reconociendo su papel en el proceso de rehabilitación de los niños y, en consecuencia, de sus cuidadores. Además, integrar técnicas lúdicas, como el juguete terapéutico, con este público, puede favorecer un ambiente acogedor y promotor de la Esperanza durante el proceso de rehabilitación. Para los cuidadores familiares, es necesario incorporar acciones de apoyo psicosocial que fortalezcan sus recursos de afrontamiento.

## DISPONIBILIDAD DE DATOS

El conjunto completo de datos en el que se basan los resultados de este estudio se ha publicado en el propio artículo.
